# The Potential of Aspen Clonal Forestry in Alberta: Breeding Regions and Estimates of Genetic Gain from Selection

**DOI:** 10.1371/journal.pone.0044303

**Published:** 2012-08-30

**Authors:** Tim Gylander, Andreas Hamann, Jean S. Brouard, Barb R. Thomas

**Affiliations:** 1 University of Alberta, Department of Renewable Resources, Edmonton, Alberta, Canada; 2 Weyerhaeuser Company Ltd., Edmonton, Alberta, Canada; 3 Isabella Point Forestry Ltd., Salt Spring Island, British Columbia, Canada; 4 Alberta-Pacific Forest Industries Inc., Boyle, Alberta, Canada; Lakehead University, Canada

## Abstract

**Background:**

Aspen naturally grows in large, single-species, even-aged stands that regenerate clonally after fire disturbance. This offers an opportunity for an intensive clonal forestry system that closely emulates the natural life history of the species. In this paper, we assess the potential of genetic tree improvement and clonal deployment to enhance the productivity of aspen forests in Alberta. We further investigate geographic patterns of genetic variation in aspen and infer forest management strategies under uncertain future climates.

**Methodology/Principal Findings:**

Genetic variation among 242 clones from Alberta was evaluated in 13 common garden trials after 5–8 growing seasons in the field. Broad-sense heritabilities for height and diameter at breast height (DBH) ranged from 0.36 to 0.64, allowing 5–15% genetic gains in height and 9–34% genetic gains in DBH. Geographic partitioning of genetic variance revealed predominant latitudinal genetic differentiation. We further observed that northward movement of clones almost always resulted in increased growth relative to local planting material, while southward movement had a strong opposite effect.

**Conclusion/Significance:**

Aspen forests are an important natural resource in western Canada that is used for pulp and oriented strandboard production, accounting for ∼40% of the total forest harvest. Moderate to high broad-sense heritabilities in growth traits suggest good potential for a genetic tree improvement program with aspen. Significant productivity gains appear possible through clonal selection from existing trials. We propose two breeding regions for Alberta, and suggest that well-tested southern clones may be used in the northern breeding region, accounting for a general warming trend observed over the last several decades in Alberta.

## Introduction

The western boreal hardwood forests cover approximately 60 million hectares, mainly in northern Alberta [1]. These resources have historically been underutilized [2]. Until the mid 80 s, conifer forest products were preferred for their superior fiber strength in both pulp and dimensional lumber products. However, this changed with advances in wood products technology, namely oriented strand board (OSB) that introduced oriented wafers of wood from poplars in combination with epoxy resins. OSB is comparable in strength to conifer plywood at a significantly lower price and today it is widely used in construction as sheeting for exterior walls, roofs, and flooring [3]. As a consequence, a sharp increase in demand for this deciduous forest resource occurred in the 1990 s and numerous oriented strand board and pulp mills were built in Alberta at this time [4]. Aspen (*Populus tremuloides* Michx.) currently represents approximately 42% of the combined conifer and deciduous annual harvest of 23 million cubic meters within the province of Alberta, making it an important local economic resource [5].

Aspen naturally grows in large, single-species, even-aged stands that regenerate clonally after fire disturbance from root suckers [6], [7]. Suckers tend to be concentrated at the distal portion of the root system, which is the reason that individual clones can expand to large areas over time [8]. This growth pattern also allows aspen to colonize marginal sites with frequent disturbances, and results in aspen clones representing arguably the oldest and largest known organisms [9], [10], [11]. Aspen could therefore be a good candidate for an intensive single-species, even-aged forest management system, and it could be argued that a clonal forestry system can be implemented that closely emulates the natural life history of aspen.

From a genetic perspective, viable tree improvement programs rely on high within-population genetic diversity for selection, and high heritability of desired traits. While previous research on aspen genetics is limited in scope, low to moderate broad-sense heritabilities of wood properties were estimated from naturally occurring aspen clones [12], [13], and moderate broad-sense heritabilities were also found in ecophysiological traits [14], [15], [16]. Molecular genetics and allozyme research with aspen has further shown very high within and among population diversity in neutral genetic markers [17], [18], [19], [20].

Traits of economic interest such as growth, survival, and insect and disease resistance are controlled by multiple genes as well as environmental factors. To distinguish environmental effects from genetic differences, genotypes are tested in common garden trials, where environmental conditions are the same for all genotypes (residual environmental variation is randomized by means of an experimental design). This allows for partitioning of the genetic variation from the overall phenotypic variation. Subsequently, the heritability of measured traits can be calculated, which represents the proportion of the total variance in a phenotypic trait that is controlled by genes. Heritability estimates can then be used to predict genetic gain from selection [21]. This type of genetic information is very limited for aspen, with only two long-term experiments reporting broad-sense heritabilities around 0.3 for height and DBH at age seven, 15, and 27 years [22], and broad-sense heritabilities around 0.6 for height and DBH in a 14-year old hybrid aspen trial [23].

In addition to estimating genetic gain from selection, knowledge about geographic structure of genetic variation is important in the development of a tree improvement program. Reforestation material needs to be well adapted to the growing conditions of the planting site, and using planting stock for reforestation that originates within a restricted geographic area can minimize loss of productivity and forest health issues because of maladaptation [24], [25], [26]. It is generally difficult to translate genetic information from common garden trials into geographic seed zones or breeding regions. However, several GIS-based techniques are now available to derive an optimal number of seed zones or breeding regions based on data from common garden trials [25], [27], [28], [29].

Recognizing the potential for an aspen tree improvement program, an industrial cooperative, the Western Boreal Aspen Cooperative (WBAC) was formed in 1995. Their initial tree improvement strategy for Alberta was originally described by Li [30], who proposed two to three breeding regions for Alberta and the establishment of a clonal test series. These trials were designed to allow for an early assessment of broad-sense heritability and convenient access to plant material for tree breeding. This paper presents a synthesis of results from the first decade of WBAC’s tree improvement program efforts, evaluating a series of 13 clonal trials to determine (1) geographic patterns of genetic variation of aspen in western Canada in order to delineate seed zones and breeding regions, and (2) to assess the potential of a clonal forestry system to enhance aspen forest productivity. The genetic data are also more generally interpreted with respect to local adaptation of aspen genotypes and forest management strategies under uncertain future climates.

## Materials and Methods

### Plus-tree Selection and Clonal Propagation

**Figure 1:**
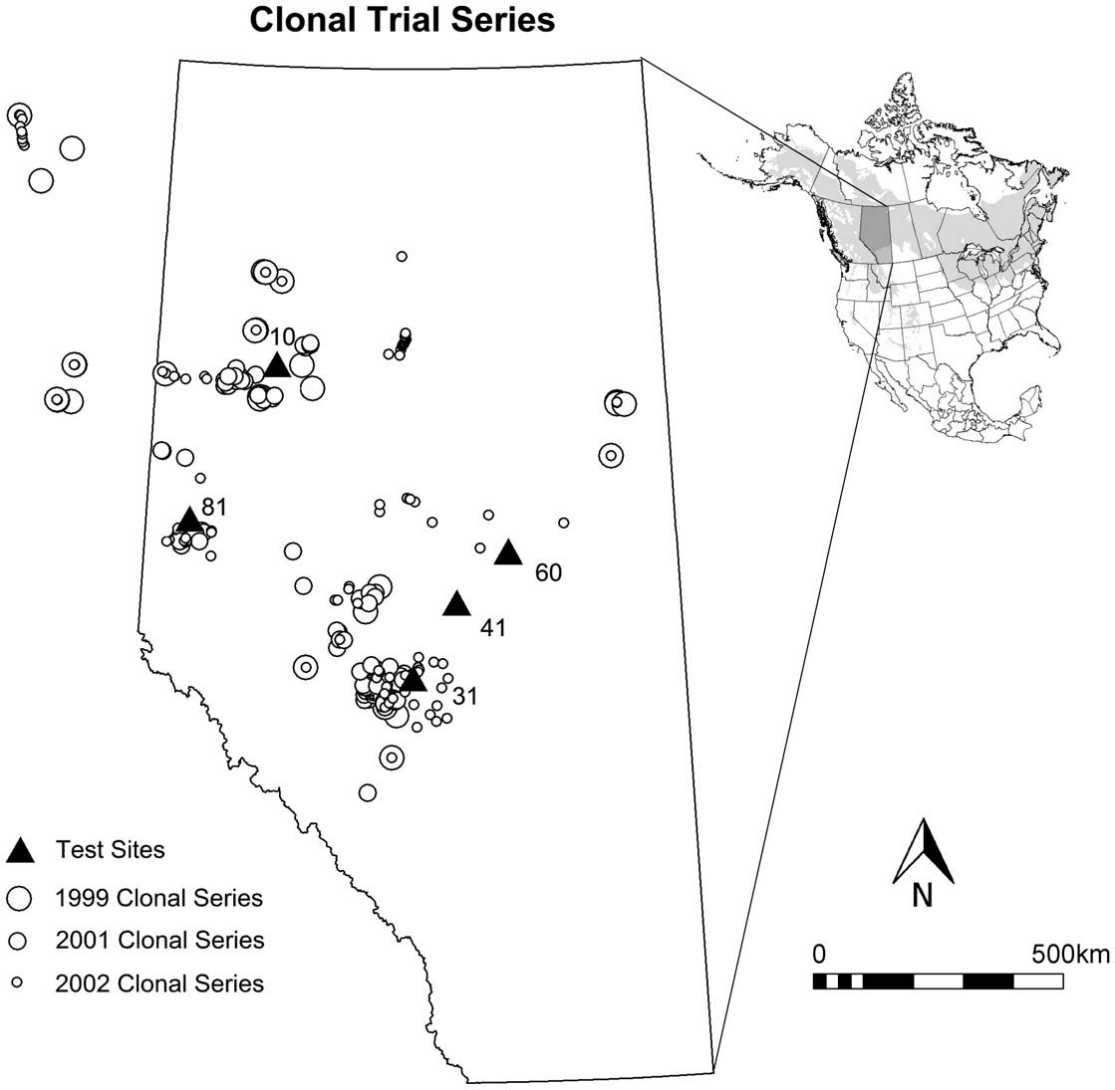
Sample sites for clone collections and test site locations of the clonal trial series. Circle sizes represent clones that were collected in different years and planted in different experiments described in Table 1. The inset shows the natural range of aspen (light gray), and the province of Alberta for orientation.

Mature trees of desirable phenotypes (plus-trees) were collected from throughout northern Alberta and north-eastern British Columbia (Figure 1), and were selected from natural stands based on good form, self-pruning in the lower half of the stem, and absence of insect and disease problems. Plus-trees were separated by a minimum of 1.5 km to ensure that they were not of the same clone. The selected trees were clonally propagated from approximately 1.5 meters of hand excavated roots with a target diameter of 2.5 cm. Root sections were collected between May and early June of three years (1998, 2000 and 2001).

**Table 1:** Site information, trial codes, and experimental design of 13 clonal trials used in this study.

				Trial codes(ID-site-year)		Number of clones	Number of blocks	Number of trees in plots
Site number and name		Site coordinates			Planted			
Code:	10	Latitude:	56°58'N	Clone1-10-99	Spring 1999	31	4	2
Name:	Manning	Longitude:	117°44'W	Clone6-10-02	Spring 2002	66	5	4
Region:	Northwestern AB	Elevation:	570 m	Clone9-10-02	Spring 2002	38	5	4
Code:	31	Latitude:	53°12'N	Clone2-31-01	Spring 2001	88	5	4
Name:	Drayton Valley	Longitude:	115°13'W	Clone3-31-01	Spring 2001	61	5	4
Region:	AB Foothills	Elevation:	887 m	Clone5-31-02	Spring 2002	77	5	4
				Clone8-31-02	Spring 2002	38	5	4
Code:	41	Latitude:	54°22'N	Clone1-41-99	Spring 1999	17	4	2
Name:	Linaria	Longitude:	114°10'W					
Region:	Central AB	Elevation:	646 m					
Code:	60	Latitude:	54°53'N	Clone1-60-99	Spring 1999	21	4	2
Name:	Athabasca	Longitude:	113°18'W	Clone18-60-01	Spring 2001	53	5	4
Region:	Central AB	Elevation:	570 m					
Code:	81	Latitude:	54°54'N	Clone4-81-01	Spring 2001	31	5	4
Name:	Grovedale	Longitude:	118°57'W	Clone7-81-02	Spring 2002	42	5	4
Region:	West-Central AB	Elevation:	683 m	Clone10-81-02	Spring 2002	36	5	4

Roots were returned to a lab and rinsed to remove soil, followed by a few minutes of a 0.5% bleaching soak for sterilization. Subsequently, 1.5 m root segments were cut into smaller pieces of approximately 30 cm length, placed into horticultural flats, covered with vermiculite and drenched with fungicide. Horticultural flats were periodically irrigated to maintain moisture content until root sprouts appeared after approximately 10 days. Once root sprouts reached 1.5 cm, they were harvested with a scalpel over a period of approximately six weeks from the horticultural flats. The sprouts were inserted into pellets of peat growth media and placed in a growth chamber at 20°C and high humidity until they rooted within one to two weeks. Rooted sprouts subsequently were transferred into larger styroblocks and grown in a nursery setting under shade for two weeks before being moved to full light in July. In the fall of the first growing season dormant root propagules (stecklings) were placed into cold storage for field planting in the subsequent spring.

### Clonal Common Garden Trials

Aspen propagules generated in the years 1999, 2001 and 2002 were planted on at least three sites established in the same year (Figure 1, Table 1). Aspen propagules were planted without regard of initial size, but they had to have a cohesive root mass filling the seedling container. The number of unique clones tested in 1999, 2001 and 2002 were 32, 112, and 118 clones, respectively. We hereafter refer to a set of clones from one year planted over multiple sites as a trial series (e.g. the 2001 trial series). Over the three years, a total of 13 clonal trials were established in randomized complete block designs. The trials varied somewhat in design and number of clones included (Table 1). Because of the large size of the experiments, two separate randomized complete block experiments were established at Site 31 for the 2001 series to accommodate all clones. Similarly, two separate randomized complete block experiments were established at sites 10, 31 and 81 for the 2002 series. Due to limited success in propagation of clonal material for the 1999 test series, this first experiment only used 2-tree row plots (Table 1).

Stecklings were planted with a spacing of approximately 2.5 meters by 3.0 meters, and a double row of border trees from leftover clonal planting material was planted around all trials. Test sites were fenced to prevent animal browse, and were managed for vegetation control comprised of a combination of chemical and mechanical means over the first three years with subsequent maintenance as needed. Measurements of height and diameter at breast height (DBH) were recorded after eight, six, and five growing seasons for the 1999, 2001, and 2002 trial series, respectively. Clonal trial series collected and established in different years had minimal or no overlap in terms of clonal material and were therefore analyzed separately.

### Data Analysis

Prior to statistical analysis, all data were examined with box plots and line plots to identify errors in measurements, data entry, or unusual growth patterns over multiple years. All trees were measured in one- or two-year intervals, and we removed all growth increments that represented a Tukey outer-fence outlier. Also, measurements identified as Tukey inner-fence outliers in one year that returned to within the interquartile range of the clone in the subsequent year were set to missing values, which was possible without detrimental effects on the statistical power of the analysis, because all treatments were replicated in multiple-tree row plots. Subsequently, individual tree data from row plots were averaged to be used as experimental units in the statistical analysis. Analysis of variance and variance component estimation was carried out separately for each site with PROC MIXED of the SAS statistical software package [31], where block and clone were specified as random factors. Average height and average DBH were calculated with the least squares means method for each experiment.

Broad-sense heritabilities were determined separately for each clonal experiment, and calculated as:
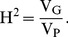
(1)where V_G_ is the total genetic variation represented by the variance component due to clone effects and V_P_ the phenotypic variation, represented by the variance component due to clones plus the residual error. Block effects were not included in the denominator. To derive standard errors for heritability estimates, standard errors of variance components were generated with the COVTEST option of PROC MIXED [31]. Using standard formulas of error propagation ([32], p. 198), the standard error of the sum of variances was determined as:



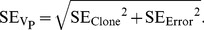
(2)Subsequently, standard formulas of error propagation for division served to estimate the standard error of H^2^:
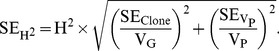
(3)


Breeding region delineations were carried out with a variance partitioning approach that used geographic predictor variables to partition genetic variation observed in common garden trials [25]. Least squares means of clones were grouped with multivariate regression tree analysis, implemented with the *MVpart* package v1.2–6 for the R programming environment [33]. Multivariate regression trees analyze multiple traits (in this case height and DBH) from multiple sites simultaneously. For each trait to be equally weighted, all variables were standardized by subtracting the mean and division by the standard deviation of each trait at each test site, so that all traits are expressed in units of standard deviations from a site mean of zero.

Multivariate regression trees (MRT) are based on the same principles as Classification and Regression Trees (CART), but extended to more than one response variable [34]. MRT can be viewed as a constrained clustering methodology that is suitable for explanation as well as prediction. A set of clusters is grown by repeated binary splits of the genetic dataset. Splits are made using predictor variables as partitioning criteria (i.e., geographic variables latitude, longitude, and elevation), so that the homogeneity of the response variable (i.e., height and DBH) within the resulting groups is maximized. Homogeneity is evaluated as sums of squares of traits around the multivariate mean of observations in a cluster [34].

## Results

### Within- and Among-population Genetic Variation

**Figure 2:**
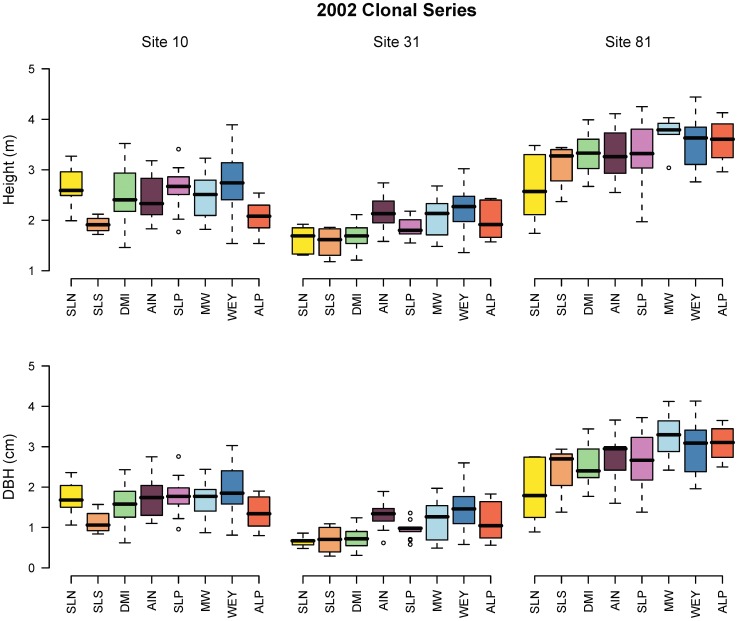
Range of aspen clone means for 5-year height and DBH at multiple test sites (the box plot indicates the range, the median, the 25th and 75th percentile of clonal means for each group. Outliers according to Tukey’s inner fence criteria are indicated by circles). The numbers of clones representing each management area are: SLN, 5; SLS, 4; DMI, 18; AIN, 9; SLP, 11; MW, 5; WEY, 18; and ALP, 5). For abbreviation of forest management areas, refer to Figure 3.

**Figure 3:**
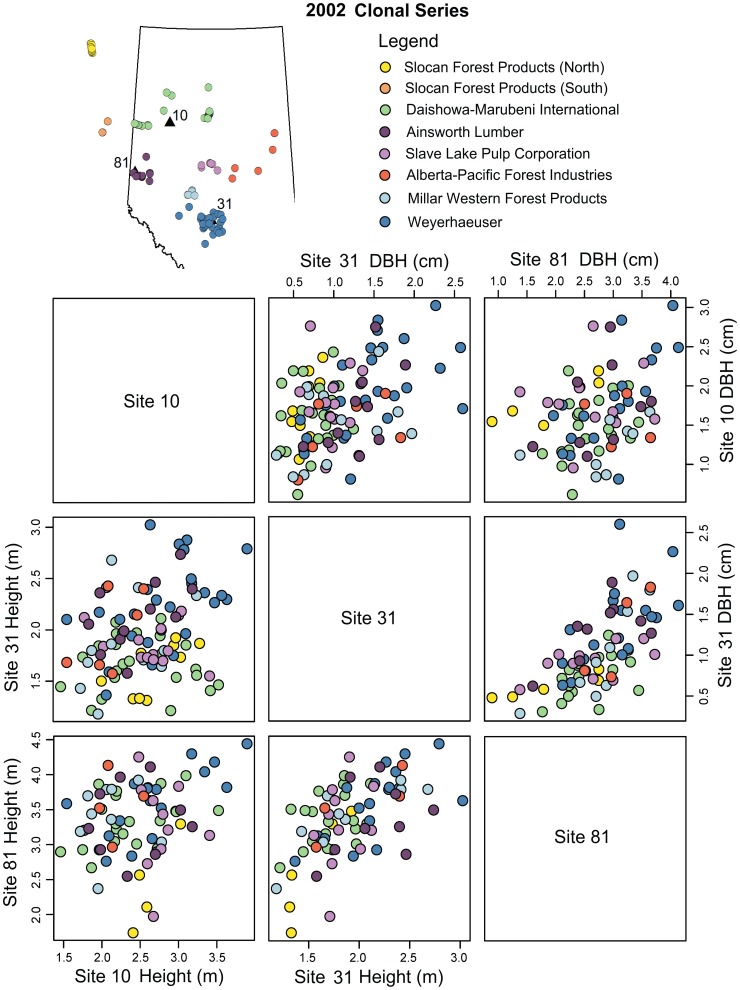
Rank changes of clones among pairs of sites for the 2002 clonal series. Scatter plots above the diagonal show 5-year DBH at two sites, and scatters below the diagonal show height. Note that in each scatter plot, only clones that were planted at both sites can be shown. The map above shows the location of test sites (triangles) and collection sites (circles). Colors represent samples from forest management areas licensed to the companies listed in the legend.

Prior to a formal variance partitioning analysis, we visualized the results of clonal trials at multiple test sites by means of box plots and scatter plots. We focus on the 2002 trial series because it contained the largest selection of clones (Figures 2 and 3), but we provide the same graphs for the 1999 and 2001 series as supporting information (Figures S1, S2, S3, and S4).

Collections in the northern forest management areas Slocan Forest Products and Daishowa-Marubeni International (SLN, SLS, and DMI) tend to perform below average at all sites, and especially poorly at the most southern Site 31 (Figure 2). The local sources from the Rocky Mountain Foothill region, especially those from the southern Weyerhaeuser forest management areas (WEY) show the highest averages as well as containing the maximum of the range of clonal means (whiskers of box plots) at all test sites.

Rank order changes of clones among pairs of test sites are shown in Figure 3. This figure represents scatter plots among all pairs of sites for DBH (above the diagonal) and for height (below the diagonal) for the 2002 trial series. Note that in each scatter plot, only clones that were planted at both sites can be shown. Rank order changes among clones are less pronounced between Site 31 and Site 81 than among Site 10 and Site 31 or Site 10 and Site 81. In two pairs that include Site 10, clones with intermediate growth change ranks frequently, resulting in broader scatters than the Site 31 and Site 81. However, note that clones exist that show superior growth at all sites (for example, in the lower left scatter plot of Figure 3, the top performer for height at Site 81 with 4.4 m (blue dot) is also a top performer at Site 10 with 3.8 m, and (moving one scatter plot up) also does well at Site 31 with a height of 2.7 m.

The 1999 and 2001 trial series reveal similar patterns. In the 1999 series (Figures S1 and S2) lower growth rates of northern sources are even more pronounced than in the 2002 series. Slocan-North sources (SLN, yellow) were the worst performers at all sites followed by the Slocan-South sources (SLS, orange), while the remaining sources do not appear to be genetically distinct in height and DBH. Despite the 2001 series (Figures S3 and S4) having a smaller geographic sampling range and fewer clones replicated across all three test sites, the results revealed similar patterns. The most northern sources from the 2001 series (DMI, green) showed the least growth at Site 31 and 81. The box plots reveal that local sources (AIN, purple) at Site 81, and (MW, WEY, shades of blue) at Site 31 performed better by a small margin.

**Figure 4:**
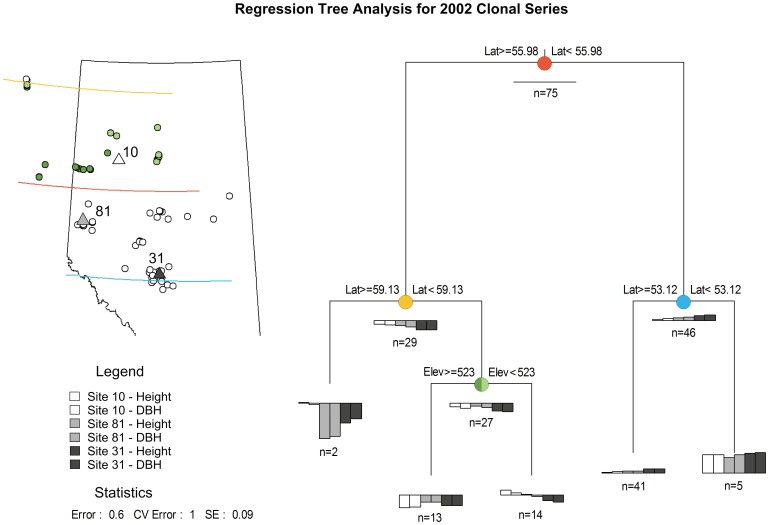
Regression tree analysis subdivides the data of the 2002 clonal series into five genetically distinct groups. Most variance in six variables (5-year DBH and height measured at three sites) is explained by three latitudinal splits at 53°, 56°, and 59°. The two very northern provenances perform very poorly at all sites except Site 10, where they show average growth. The five most southern sources perform above average at all sites. The 13 sources from the boreal highlands (≥523 m) perform somewhat below average at all sites while the 14 northern lower elevation sources perform slightly above average at Site 10 and slightly below average at Site 31. A large group of 41 sources from the foothills and central Alberta shows a wide range of performance, but no geographic patterns of genetic variation.

Figure 4 shows the results of variance partitioning for the 2002 clonal trial series using the multivariate regression tree approach. The 2002 clonal series was chosen because of the wide geographic coverage and representation of all clones at all test sites. The amount of genetic variation explained by each split of the dataset is represented by the length of the branches. Most variation within the 2002 clonal dataset (height and DBH at three sites) can be explained by a split at approximately 56°N latitude. Further splits separate the five most southern sources, which perform above average at all sites (Figure 4, group on the far right). The next split separates the two most northern sources in BC, which perform far below average at Sites 31 and 81 (group on far left). The last minor split separates sources from above and below 523 m within the northern Alberta group.

### Broad-sense Heritability of Growth Traits

Based on the regional split presented above, identifying two breeding regions for Alberta separated at approximately 56°N latitude, we separately analyzed northern clones at the northern site (Slocan and DMI sources planted at Site 10) and southern clones at the southern planting sites (all other sources at Sites 31, 41, 81, and 60). Variance components and heritabilities for these putative breeding populations are provided in Tables 2 and 3.

**Table 2:** Number of clones included in trials, mean height and DBH of trials, variance components, and heritability estimates with standard errors (SE) for southern breeding region trials.

	Trial code(ID-site-year)	Numberof clones	Trialmean	Variance components							
Variable				Clone (SE)		Block (SE)		Error (SE)		H^2^ (SE)	
Height (m)	Clone1-41-99	4	5.71	0.13	(0.20)	0.37	(0.40)	0.40	(0.20)	0.24	(0.39)
	Clone1-60-99	6	4.91	0.56	(0.45)	0.14	(0.20)	0.52	(0.20)	0.52	(0.48)
	**Clone2-31-01**	**77**	**3.14**	**0.19**	**(0.04)**	**0.03**	**(0.02)**	**0.14**	**(0.01)**	**0.58**	**(0.13)**
	Clone3-31-01	46	2.82	0.21	(0.05)	0.03	(0.02)	0.17	(0.02)	0.55	(0.16)
	Clone4-81-01	20	4.76	0.30	(0.14)	0.11	(0.10)	0.58	(0.10)	0.35	(0.17)
	Clone18-60-01	53	3.51	0.38	(0.09)	0.01	(0.02)	0.31	(0.04)	0.55	(0.16)
	**Clone5-31-02**	**51**	**1.96**	**0.11**	**(0.03)**	**0.02**	**(0.02)**	**0.10**	**(0.01)**	**0.51**	**(0.14)**
	Clone7-81-02	28	3.28	0.23	(0.09)	0.19	(0.15)	0.33	(0.05)	0.41	(0.17)
	**Clone8-31-02**	**24**	**2.23**	**0.13**	**(0.04)**	**0.04**	**(0.03)**	**0.10**	**(0.02)**	**0.56**	**(0.22)**
	Clone10-81-02	22	3.73	0.11	(0.05)	0.11	(0.09)	0.28	(0.05)	0.27	(0.15)
DBH (cm)	Clone1-41-99	4	5.87	0.00		0.59	(0.64)	0.67	(0.29)	0.00	
	Clone1-60-99	6	6.20	0.91	(0.77)	0.00		0.92	(0.33)	0.50	(0.48)
	**Clone2-31-01**	**77**	**2.60**	**0.51**	**(0.10)**	**0.10**	**(0.07)**	**0.37**	**(0.03)**	**0.58**	**(0.13)**
	Clone3-31-01	46	2.18	0.40	(0.11)	0.10	(0.08)	0.41	(0.05)	0.49	(0.15)
	Clone4-81-01	20	4.45	0.64	(0.27)	0.17	(0.15)	0.89	(0.15)	0.42	(0.19)
	Clone18-60-01	53	3.61	0.62	(0.16)	0.03	(0.03)	0.60	(0.08)	0.51	(0.15)
	**Clone5-31-02**	**51**	**1.15**	**0.19**	**(0.05)**	**0.03**	**(0.03)**	**0.19**	**(0.02)**	**0.50**	**(0.14)**
	Clone7-81-02	28	2.71	0.36	(0.13)	0.28	(0.22)	0.55	(0.08)	0.39	(0.16)
	**Clone8-31-02**	**24**	**1.37**	**0.22**	**(0.07)**	**0.05**	**(0.04)**	**0.12**	**(0.02)**	**0.64**	**(0.25)**
	Clone10-81-02	22	3.15	0.18	(0.09)	0.20	(0.16)	0.52	(0.09)	0.25	(0.14)

Note that only clones and test sites south of 56°N latitude were included. The trials most promising for selecting superior clones (with the highest heritabilities and most clones within a series) are highlighted in bold.

**Table 3:** Number of clones included in trials, mean height and DBH of trials, variance components, and heritability estimates with standard errors (SE) for northern breeding region trials.

	Trial code(ID-site-year)	Numberof clones	Trialmean	Variance components							
Variable				Clone (SE)		Block (SE)		Error (SE)		H^2^ (SE)	
Height (m)	**Clone1-10-99**	**19**	**4.33**	**0.13**	**(0.07)**	**0.02**	**(0.03)**	**0.24**	**(0.05)**	**0.36**	**(0.21)**
	**Clone6-10-02**	**23**	**2.63**	**0.12**	**(0.04)**	**0.11**	**(0.08)**	**0.11**	**(0.02)**	**0.52**	**(0.22)**
	Clone9-10-02	14	2.15	0.02	(0.02)	0.00	(0.01)	0.14	(0.03)	0.13	(0.13)
DBH (cm)	**Clone1-10-99**	**19**	**4.14**	**0.58**	**(0.25)**	**0.00**		**0.50**	**(0.10)**	**0.54**	**(0.27)**
	**Clone6-10-02**	**23**	**1.63**	**0.11**	**(0.04)**	**0.07**	**(0.06)**	**0.13**	**(0.02)**	**0.46**	**(0.19)**
	Clone9-10-02	14	1.35	0.06	(0.03)	0.03	(0.03)	0.15	(0.03)	0.27	(0.18)

Note that only clones and test sites north of 56°N latitude were included. The trials most promising for selecting superior clones (with the highest heritabilities and most clones within a series) are highlighted in bold.

Broad-sense heritabilities for height and DBH for the southern breeding region and southern collections by Weyerhaeuser, Millar Western Forest Products, Alberta-Pacific Forest Industries Inc., Ainsworth Lumber, and Slave Lake Pulp Corporation were variable among individual trials (Table 2). Generally, Site 31 at Drayton Valley and Site 60 at Athabasca showed high heritability values from 0.50 to 0.64. These were high-quality sites that were well maintained with uniform planting conditions. Given that Site 31 also contains the most clones within annual series, experiments at Site 31 (highlighted in bold in Table 2) appear to be the most promising to select from for breeding and deployment. After removal of northern clones, the 1999 clonal trials only contained four to six clones, which were not sufficient for accurate estimation of heritability in this series.

The northern collections by Slocan Forest Products and Daishowa-Marubeni International at the northern Site 10 yielded somewhat lower broad-sense heritabilities between 0.13 and 0.54, for height and DBH. Standard errors in this trial series were generally higher and the number of clones included in the trials was generally lower. Nevertheless, two experiments (highlighted in bold in Table 3) appear to be suitable to select clones from for breeding and clonal deployment with a total number of 42 clones.

## Discussion

### Delineation of Breeding Regions

The analysis of geographic patterns of genetic variation confirms Li’s [30] preliminary delineation of breeding regions for Alberta. In fact, his educated guess of three breeding regions for Alberta, which was based on very limited genetic information, appears remarkably insightful. His proposed north-south split at 55°N latitude, corresponds almost exactly to our proposed 56°N latitude splits determined by regression tree analysis. In a previous study with wider geographic sampling covering western Canada and Minnesota but a lower sampling density Hamann et al. [25] obtained a similar result with different (seedling based) aspen collections. Also in our current study, we could not detect an east-west differentiation of aspen genotypes that would justify a third breeding region east of 114°W longitude for Alberta, also proposed by Li [30]. Our findings also conform to *in situ* observations by Barnes [35], who identified a significant north-south cline in morphology of aspen, but not an east-west differentiation.

Regression tree analysis for the 2002 clonal trial series also identified an elevational differentiation of genotypes within the region north of 56°N latitude, which could not be detected by Hamann et al. [25], due to low regional sampling density. Sources from above 500 m within this region consistently underperformed across all test sites in the clonal trial series, which suggests that there should be an elevation limit to the northern breeding region. While we did not find an elevational cline in the southern breeding region, no high elevation clones were included in the test series, and a cautious recommendation may therefore limit the breeding region to the highest sampled clones until appropriate data become available.

### Potential Gains from Deployment of Clonal Aspen

Across 13 clonal trials, broad-sense heritabilities in this study were on average 0.45 for height and 0.43 for DBH. The trials with the highest heritabilities and largest numbers of clones (highlighted in Tables 2 and 3), indicate considerable potential for achieving genetic gains through selecting superior clones for deployment prior to a first generation breeding cycle as a tree improvement strategy. The results also highlight the importance of carefully selecting uniform sites and applying methodical vegetation management. Trials where this was not logistically possible in our study (not highlighted in Table 2 and 3) are essentially a lost investment with respect to selection and tree breeding.

To estimate genetic gains from selection, we have to determine selection differentials that can be achieved with the current trial series. High selection differentials require a large base population, which can be increased by combining clones planted at different sites. This is, strictly speaking, not possible in our case, because there are not enough common clones among experiments for a joint analysis (at most, four clones overlapped among experiments belonging to separate series. Often, there was no overlap). However, for an informal assessment, we can rank clones across different genetic tests by expressing height and DBH in units of standard deviations from a test mean of zero. For the following assessment we therefore make the assumption that the average genetic worth of different clonal trial series is the same, and that the effectiveness of plus-tree selections was similar in different years.

Clonal deployment of planting stock in Alberta requires at least 18 clones in a deployment population for reforestation on public lands [5]. For the southern breeding region, 146 southern clones can be assessed at Site 31, allowing for a selection differential of 12% when selecting the top 18 clones. This results in a 15% genetic gain in height and 34% genetic gain in DBH, assuming an average broad-sense heritability at the selected sites (highlighted in Tables 2) of 0.55 and 0.57, for height and DBH respectively. The northern breeding region has fewer clones available at Site 10, with 42 northern clones. (We excluded the Clone9-10-02 experiment because of low heritabilities (Table 3)). Forty two clones only allow for a selection differential of 43% when selecting the top 18 clones. This results in a 5% genetic gain in height and 9% genetic gain in DBH, assuming an average broad-sense heritability at the selected sites (highlighted in Tables 2 and 3) of 0.44 and 0.5 for height and DBH, respectively.

### Local Optimality of Seed Sources

The conceptual basis for the delineation of breeding regions is that local sources are optimally adapted to local environments and should therefore not to be moved too far from the collection location to avoid mal-adaptation. Our results suggest that this assumption may not apply for aspen in Western Canada. Instead, a northward movement of planting material almost always resulted in increased growth and a southward movement had a strong opposite effect. Northern sources (SLN, SLS, and DMI) generally underperformed at southern sites (31, 41, and 81), whereas more southern sources (WEY, ALP, MW, AIN) generally performed better or on-par with local material at the northern site (Figures 3, S1, and S3). A likely explanation for this observation is adaptational lag, which refers to a mismatch of genotypes and environments caused by a relatively fast environmental change and a comparably slow evolutionary response. This is a plausible explanation in light of substantial environmental changes towards drier and warmer climate conditions during the test period, when compared to long-term climate normal conditions for the past century [36], [37].

We should note, however, that the observation of local non-optimality is not uncommon, and may have a number of natural causes besides adaptational lag [26], [38], [39]. Founder effects, at the northern range limit and subsequent persistence of genotypes through clonal regeneration could be a plausible explanation for local sub-optimality relative to populations transferred from more southern origins [26]. Asymmetric gene flow from the center to peripheral populations may also cause sub-optimality [40], although this mechanism would result in growth patterns opposite to those observed here. A more plausible alternative explanation may be an apparent non-optimality, as evolutionary fitness is not necessarily reflected by growth measured in short-term common garden trials. Instead, a evolutionary fitness may require investment of plant resources in adaptive traits that only become relevant under rare climatic extreme events [39].

If southern clones from the 1999 to 2002 series, tested at the northern site (10) were used in the northern breeding region, the top 18 clones would comprise six northern sources and 12 southern sources, yielding a 16% genetic gain in height. The additional 11% gain may be associated with an increased risk, depending on the underlying cause of the observed non-optimality. If adaptational lag is the underlying cause, the use of well tested southern sources in the northern breeding region would be a sensible climate change adaptation strategy. If reduced growth of local sources is caused by tradeoffs required by survival adaptations, those genetic gains could be associated with some risks. For example frost damage may occur under rare, unseasonal cold events. After only 5–8 growing seasons in the field, we found no indication of such tradeoffs. Survival was generally high, and survival of southern clones transplanted to the northern test sites (99%) was nearly identical to that of local sources at the northern trials (98%, data not shown). Nevertheless, a cautious approach to seed transfer would avoid long-distance relocations, exceeding 2–3° latitude.

## Conclusions and Recommendations

Two breeding regions for aspen in Alberta, north and south of 56°N latitude are supported by our results. It should be noted, however, that none of the clones sampled in Alberta were collected north of 59°N latitude and this should therefore be a prudent limit for the northern breeding region. Similarly no material was tested south of 52°30′N, which would serve as a limit for the southern breeding region. The northern breeding region may include an elevation limit, since we detected a genetic difference along an elevational gradient in the 2002 clonal trial series. However, given observed warming trends in Alberta this limit should mainly be viewed as a restriction against including higher elevation sources for low elevation deployment, not vice versa. Data from this and a previous study further suggests that eastern and western regions of Alberta at the same latitude and similar ecosystem types do not require separate breeding regions for lack of genetic differentiation.

Broad-sense heritabilities estimated from multiple trials suggest that substantial genetic gains are possible through clonal selection as part of an aspen tree improvement program. Broad-sense heritabilities for height and DBH ranged from 0.36 to 0.64 on good sites. In a first round of selection for the southern breeding region, 15% genetic gain in height and 34% in DBH could be achieved immediately through deployment of clones, prior to the first generation of breeding. For the northern breeding region, genetic gains are predicted to be smaller (5% for height and 9% in DBH), because of lower heritabilities and fewer clones currently available for selection. However, including southern sources that have been tested at the northern site would also result in a 16% genetic gain for the northern breeding region as well. Local non-optimality observed in this study and recent climate change trends observed in Alberta suggests that in general transfer of any genetic material toward lower elevation and towards the south should be avoided, while upward and northward transfer of genetic material should be part of a tree improvement strategy to achieve enhanced forest productivity in the future.

## Supporting Information

Figure S1 **Range of aspen clone means for 8-year height and DBH at multiple test sites (the box plot indicates the range, the median, the 25th and 75th percentile of clonal means for each group.** Outliers according to Tukey’s inner fence criteria are indicated by circles). The total numbers of clones representing each management area are: SLN, 10; SLS, 4; DMI, 25; AIN, 11; SLP, 14; MW, 15; WEY, 32; and ALP, 7). For abbreviation of forest management areas, refer to Figure S2.
(PDF)

Figure S2 **Rank changes of clones among pairs of sites for the 1999 clonal series.** Scatter plots above the diagonal show 8-year DBH at two sites, and scatters below the diagonal show height. Note that in each scatter plot, only clones that were planted at both sites can be shown. The map above shows the location of test sites (triangles) and collection sites (circles).
(PDF)

Figure S3 **Range of aspen clone means for 6-year height and DBH at multiple test sites (the box plot indicates the range, the median, the 25th and 75th percentile of clonal means for each group.** Outliers according to Tukey’s inner fence criteria are indicated by circles). The total numbers of clones representing each management area are: DMI, 23; AIN, 14; MW, 9; and WEY, 66). For abbreviation of forest management areas, refer to Figure S4.
(PDF)

Figure S4 **Rank changes of clones among pairs of sites for the 2001 clonal series.** Scatter plots above the diagonal show 6-year DBH at two sites, and scatters below the diagonal show height. Note that in each scatter plot, only clones that were planted at both sites can be shown. The map above shows the location of test sites (triangles) and collection sites (circles).
(PDF)
