# Supplementary material for: The Potential of Aspen Clonal Forestry in Alberta: Breeding Regions and Estimates of Genetic Gain from Selection
Source: PLoS One. 2012 Aug 30;7(8):e44303. doi: 10.1371/journal.pone.0044303 (PMC3431317; doi:10.1371/journal.pone.0044303)
Supplement: Figure S3 — Range of aspen clone means for 6-year height and DBH at multiple test sites (the box plot indicates the range, the median, the 25th and 75th percentile of clonal means for each group. Outliers according to Tukey’s inner fence criteria are indicated by circles). The total numbers of clones representing each management area are: DMI, 23; AIN, 14; MW, 9; and WEY, 66). For abbreviation of forest management areas, refer to Figure S4. (PDF) [file pone.0044303.s003.pdf]

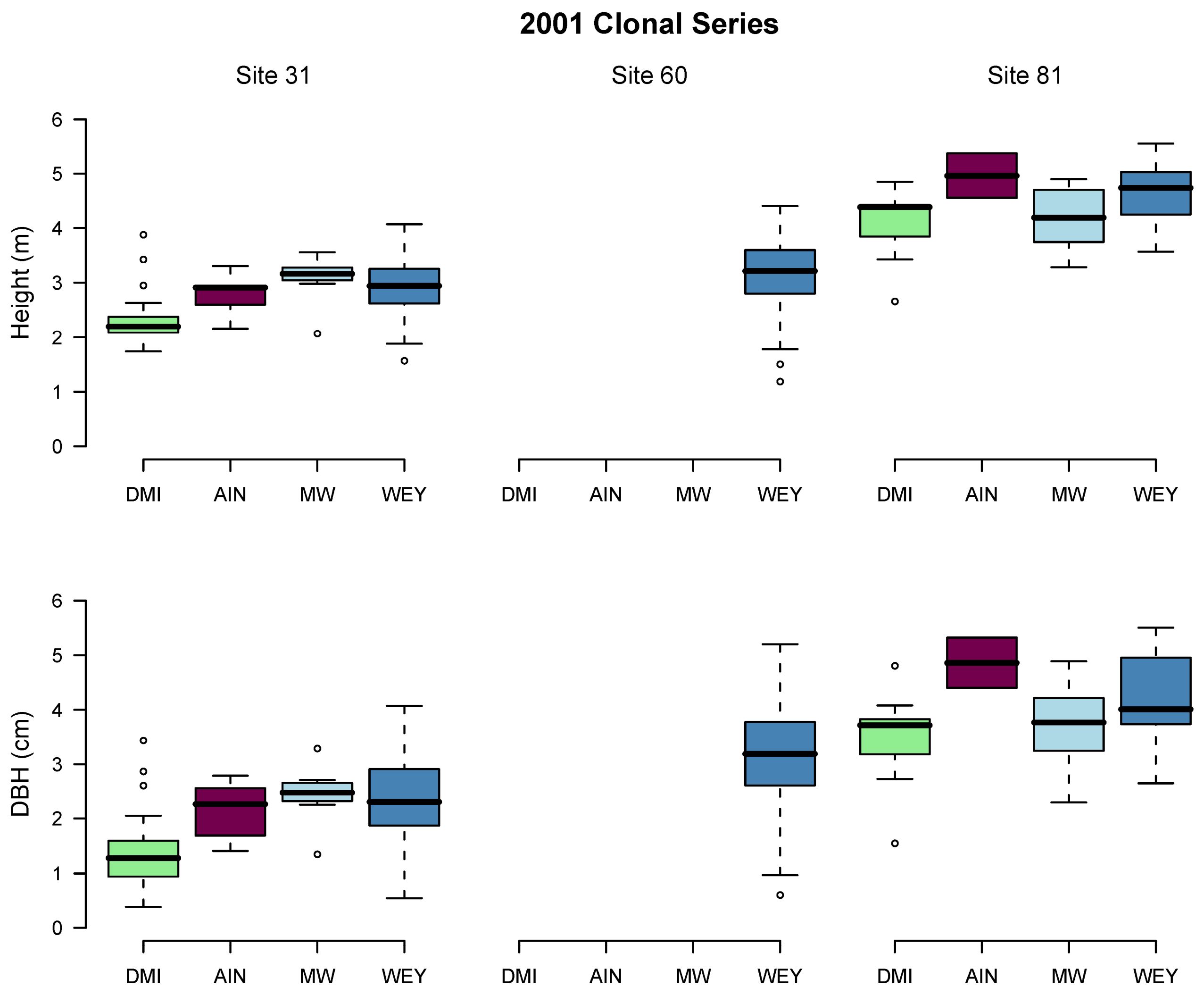

Figure S3. Range of aspen clone means for 6-year height and DBH at multiple test sites (the box plot indicates the range, the median, the 25th and 75th percentile of clonal means for each group. Outliers according to Tukey's inner fence criteria are indicated by circles). The total number of clones representing each management area are: DMI, 23; AIN, 14; MW, 9; and WEY, 66). For abbreviation of forest management areas, refer to Figure S4.
