# Supplementary material for: The Potential of Aspen Clonal Forestry in Alberta: Breeding Regions and Estimates of Genetic Gain from Selection
Source: PLoS One. 2012 Aug 30;7(8):e44303. doi: 10.1371/journal.pone.0044303 (PMC3431317; doi:10.1371/journal.pone.0044303)
Supplement: Figure S4 — Rank changes of clones among pairs of sites for the 2001 clonal series. Scatter plots above the diagonal show 6-year DBH at two sites, and scatters below the diagonal show height. Note that in each scatter plot, only clones that were planted at both sites can be shown. The map above shows the location of test sites (triangles) and collection sites (circles). (PDF) [file pone.0044303.s004.pdf]

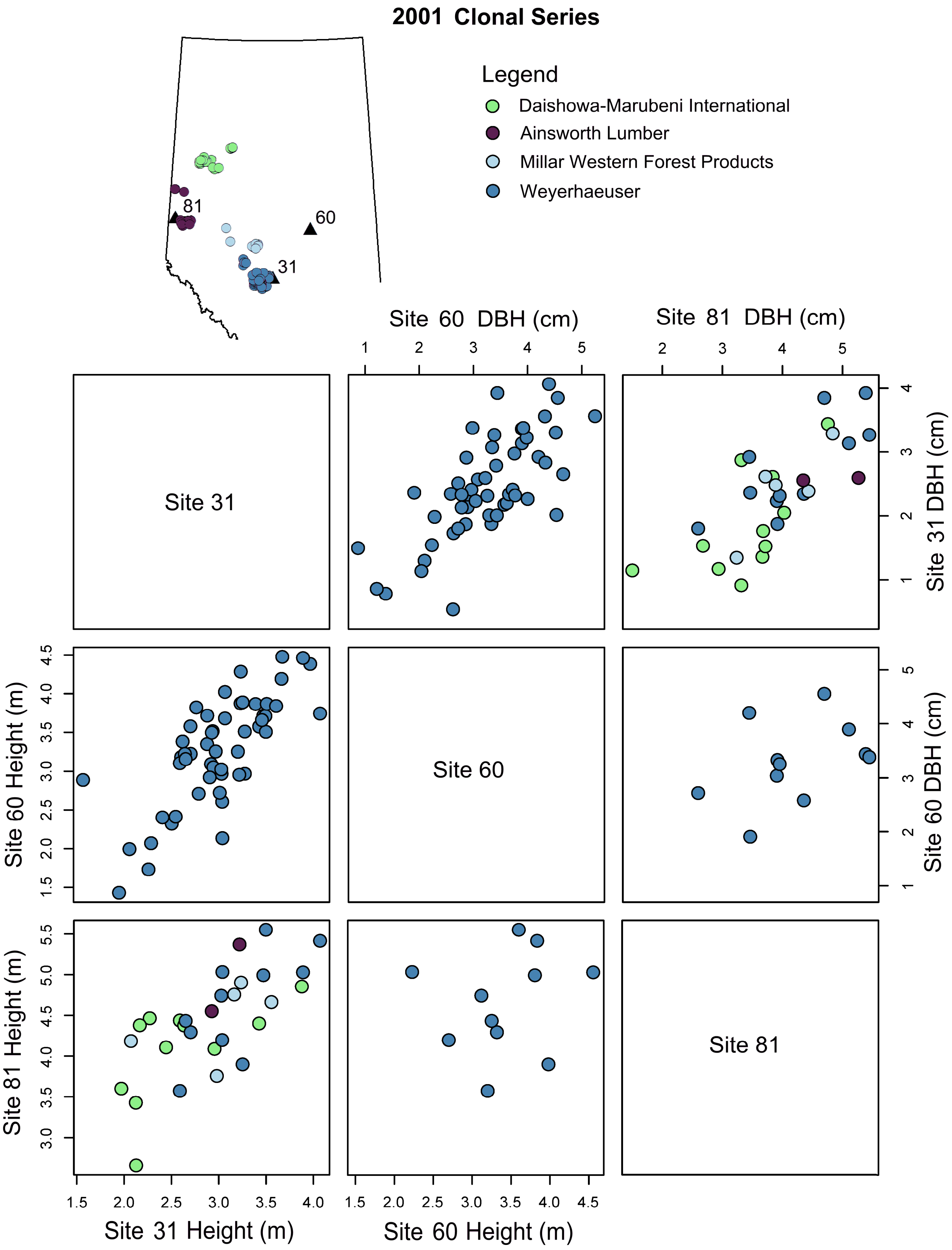

Figure S4. Rank changes of clones among pairs of sites for the 2001 clonal series. Scatter plots above the diagonal show 6-year DBH at two sites, and scatters below the diagonal show height. Note that in each scatter plot, only clones that were planted at both sites can be shown. The map above shows the location of test sites (triangles) and collection sites (circles).
